# Effects of Erchen Decoction on Oxidative Stress-Related Cytochrome P450 Metabolites of Arachidonic Acid in Dyslipidemic Mice with Phlegm-Dampness Retention Syndrome: A Randomized, Controlled Trial on the Correspondence between Prescription and Syndrome

**DOI:** 10.1155/2022/1079803

**Published:** 2022-03-29

**Authors:** Jing Chen, Chao Ye, Zheng Yang, Pinhui Li, Hongfei Wu, Bing Xu, Shan Zhang, Xiaolin Xue

**Affiliations:** ^1^Preventive Treatment of Disease Department, The Third Affiliated Hospital, Beijing University of Chinese Medicine, Beijing 100029, China; ^2^Orthopedics Department, Dongzhimen Hospital, Beijing University of Chinese Medicine, Beijing 100700, China; ^3^School of Traditional Chinese Medicine, Beijing University of Chinese Medicine, Beijing 100029, China; ^4^Cardiovascular Department, Hainan Provincial Hospital of Traditional Chinese Medicine, Haikou, Hainan, China; ^5^Traditional Chinese Medicine Department, Tibetology Research Center of Beijing Tibetan Medicine Hospital, Beijing, China

## Abstract

Phlegm-dampness retention (PDR) syndrome is one of the main syndromes of dyslipidemia. This study investigated the effects of Erchen decoction (ECD) on concentrations of two oxidative stress-related cytochrome P450 (CYP450) metabolites of arachidonic acid—14,15-dihydroxyeicosatrienoic acid (14,15-DHET) and 20-hydroxyeicosatetraenoic acid (20-HETE)—in mice with dyslipidemia and phlegm-dampness retention (PDR) syndrome (*n* = 5 C57BL/6J mice and *n* = 30 apolipoprotein E knockout mice). Murine models of the disease and syndrome were established using multifactor stimulation. Then, all mice were assigned to normal, model, low- (L-), medium- (M-), or high- (H-) dose ECD groups or to a control or an unmatched prescription-syndrome (unmatched P-S) group; five mice were included in each group. Dose formulations were administered by oral gavage for 30 days to animals in the corresponding groups. We detected and analyzed hematoxylin and eosin (HE) staining characteristics of the mouse aorta and serum total cholesterol (TC), low-density lipoprotein cholesterol (LDL-C), peroxynitrite (ONOO^−^), 14,15-DHET, and 20-HETE concentrations in each group. TC and LDL-C concentrations significantly decreased in the M-ECD versus control group (*P* < 0.05); however, the TC and LDL-C concentrations were not significantly different in the unmatched P-S versus model group (*P* > 0.05). After treatment in the P-S correspondence groups (L-ECD, M-ECD, and H-ECD groups), the concentration of ONOO^−^ decreased to different degrees in each group. Among these groups, the concentration of ONOO^−^ significantly decreased in the L-ECD, M-ECD, and H-ECD groups versus the model group (*P* < 0.05). However, the concentration of ONOO^−^ was not significantly different in the unmatched P-S versus the model group (*P* > 0.05). From the perspective of aortic HE staining, the P-S group experienced an improved endothelium structure after treatment. 14,15-DHET concentrations significantly increased in the normal, M-ECD, and H-ECD groups versus the model group; in the H-ECD versus the L-ECD group; and in the H-ECD versus the control group (all *P* < 0.05) to various extents after different doses of the prescription. 20-HETE concentrations pronouncedly decreased in the M-ECD versus normal group; in the M- and H-ECD groups versus the model group; in the M-ECD versus the L-ECD group; in the M-ECD versus the control group; and in the M-ECD versus the unmatched P-S (*P* < 0.05). However, the concentrations of 14,15-DHET and 20-HETE in the model group were not significantly different from those of the unmatched P-S (*P* > 0.05). In this study, ECD reversed blood lipid indexes and ameliorated oxidative stress-related metabolites, elevating serum 14,15-DHET and lowering serum 20-HETE in mice with dyslipidemia and PDR syndrome via CYP450 pathways of arachidonic acid metabolism. The efficacy of ECD relies on correspondence between the prescription and the syndrome. These findings scientifically validate the treatment according to traditional Chinese medicine syndrome differentiation. ECD can strengthen the protective effect on the vascular endothelium by driving out pathogenic factors and strengthening healthy resistance. Its efficacy may be related to the adjustment of the polarization state of macrophages.

## 1. Introduction

Dyslipidemia has become more prevalent among Chinese adults during recent years as a result of better living conditions [1]. The condition may elevate serum oxygen free radical levels and exacerbate the inflammatory response, leading to vascular endothelial cell damage [2] that damage in turn has close associations with cardiocerebrovascular diseases, such as coronary atherosclerotic heart disease and cerebral infarction [3]. Macrophage recruitment around endothelial cells is essential during vascular endothelial cell damage and is an early cause of plaque formation [4].

Traditional Chinese medicine (TCM) treatments have multitarget, multipathway, and multidimensional features. The efficacy of TCM in treating dyslipidemia has been demonstrated [5]. Herbs used in TCM herbs contain various active components, and their properties are often categorized according to TCM theory into four natures (hot, warm, cool, and cold), five flavors (salty, sweet, sour, spicy, and bitter), and medical actions of ascending, descending, floating, and sinking. The selection of proper herbs for patients boils down to an accurate diagnosis of TCM syndromes, which underlies the efficacy of TCM treatment. The characteristics of phlegm-dampness retention (PDR) syndrome, which is frequently diagnosed in patients with dyslipidemia, have been the focus of exploration in our study series.

One important mechanism of endothelial dysfunction is the enhancement of oxidative stress. Excess nitric oxide (NO) can combine with superoxide to form peroxynitrite (ONOO^−^). ONOO^−^ causes oxidative stress and tissue damage in the vascular endothelium through protein oxidation or nitration. Our research group previously found that the serum concentration of ONOO^−^ increased in patients with dyslipidemia and PDR syndrome; in fact, its concentration can reflect the degree of PDR syndrome [6]. This research showed that enhanced oxidative stress is a characteristic of PDR syndrome with dyslipidemia. Therefore, studying the PDR syndrome and dyslipidemia by assessing the characteristics of oxidative stress is feasible.

Macrophages, including M1 and M2 subtypes [7], are critical for vascular endothelial injury in dyslipidemia. M1 macrophages are responsible for inflammatory factor release to fuel inflammation, inducing vascular endothelial cell damage [8]; conversely, M2 macrophages generate anti-inflammatory mediators involved in angiogenesis and tissue growth, thus limiting the severity of inflammation [9]. The two functionally antagonistic phenotypes can be transformed into each other under various stimulations; this transformation is consistent with the struggle between or waxing and waning of healthy qi (energy) and pathogenic factors during the onset and progression of diseases and syndromes, as described in TCM theory. In a previous study series, we established murine models of dyslipidemia with PDR syndrome and spleen–kidney yang deficiency (SKYD) syndrome using multifactor stimulation that can mimic the core physiopathological process of the disease and simulate the critical pathogenesis of the syndrome. RNA sequencing of endothelial macrophages has revealed that increased levels of CYP450 metabolites of arachidonic acid are a predominant feature of dyslipidemia with PDR [10]. CYP450 pathways are one pathway that drives arachidonic acid metabolism [11], and the metabolites may impair endothelial cell function through oxidative stress. The molecules 14,15-dihydroxyeicosatrienoic acid (14,15-DHET) and 20-hydroxyeicosatetraenoic acid (20-HETE) are critical downstream molecules in the CYP450 pathway of arachidonic acid metabolism [12].

Prescription-syndrome (P-S) correspondence often uses counterevidence for the prescription-syndrome relationship from drugs, formulas, treatment strategies, and prescription disciplines; in P-S, formulas must be prescribed for the syndrome and adjusted with the syndrome. The molecular changes that occur during matched P-S treatment can be the material basis of the syndrome. Erchen decoction (ECD) is a classic TCM formula for PDR syndrome described in *Prescriptions of Peaceful Benevolent Dispensary* [13]; it is a matched prescription for PDR with dyslipidemia because the prescription discipline conforms to the syndrome pathogenesis. However, whether the blood lipid index, aortic endothelial structure, serum ONOO-, CYP450 metabolites, 14,15-DHET, or 20-HETE can be altered by treatment remains unknown. Therefore, we explored the effects of ECD on these variables in mice with dyslipidemia and PDR syndrome.

In this study, we used apolipoprotein E knockout (ApoE-/-) mice to generate models of dyslipidemia with PDR or SKYD for matched and unmatched prescription-syndrome experiments. ECD was used as the intervention to explore its effect on the serum total cholesterol (TC), low-density lipoprotein cholesterol (LDL-C), ONOO^−^, 14,15-DHET, and 20-HETE levels. The objectives of this study are threefold: (1) to investigate whether ECD affected oxidative stress-related molecules in dyslipidemia with PDR syndrome via CYP450 pathways of arachidonic acid metabolism; (2) to scientifically validate the treatment according to TCM syndrome differentiation from the perspective of prescription-syndrome correspondence; and (3) to speculate about the correlation of the efficacy of ECD with macrophage polarization according to characteristic changes in CYP450 metabolites of arachidonic acid as a key player in dyslipidemia with PDR syndrome.

## 2. Materials and Methods

**Figure 1:**
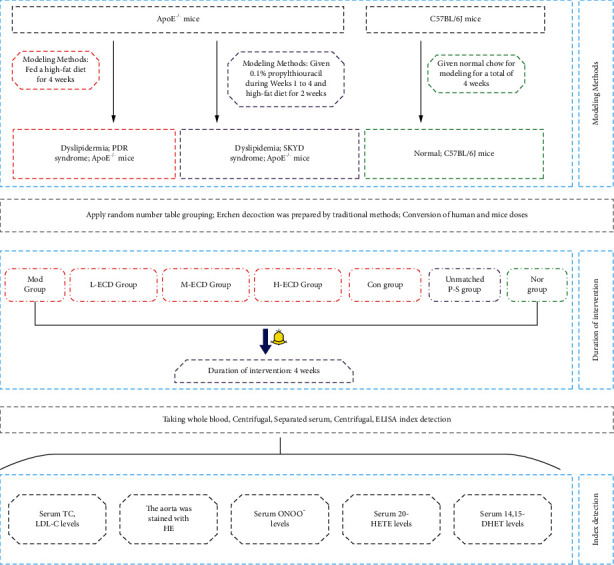
Research flowchart.

Figure 1 presents an overview of the study's materials and methods, described in full as follows.

### 2.1. Experimental Animals

The animals featured in the research included 30 ApoE-/- mice; all were male, were 6 weeks old, and had a body mass of approximately 20 (±5) g. In addition, five C57BL/6J mice of the same strain were used; all were male, were 6 weeks old, and had a body mass of approximately 20 (±5) g. All animals were raised in the Beijing Changyang Xishan Farm; the license number for the use of experimental animals is SCXK (jing) 2019-0008. The rearing environment incorporated a room temperature of 21–25°C, 50%–70% humidity, and 12 h of alternating light and shade.

All procedures in this study followed the *NIH Guide for the Care and Use of Laboratory Animals* (revised 2011). This study was approved by the Medical and Experimental Animal Ethics Committee of Beijing University of Chinese Medicine (approval no. BUCM-4-2021090102-3098; Beijing, China).

### 2.2. Drugs and Preparations

Erchen decoction is composed of six kinds of Chinese herbal medicine: pinelliae praeparata (fabanxia), 15 g; poria (fuling), 15 g; pericarpium citri reticulatae (chenpi), 10 g; glycyrrhizae radix et rhizoma (zhigancao), 6 g; zingiberis rhizoma recens (shengjiang), 10 g; and mume fructus (wumei), 3 g.

All Chinese herbal medicines were provided by The Third Affiliated Hospital, Beijing University of Chinese Medicine. The Chinese herbal medicine decoction was prepared according to the conventional TCM decoction method: (1) all herbs were placed into a saucepan (porcelain) with 500 mL of water; (2) after the herbs were soaked for 30 min, they were boiled over high heat; (3) the heat was reduced to low and the decoction simmered for 20 min; (4) the liquid was poured out; (5) water was added again, brought to a boil over high heat, then reduced to low heat and simmered for 15 min; (6) concentrate the two solutions by putting them together.

Atorvastatin calcium tablets were used as a positive control (Meidaxin, 10 mg/tablet; Qilu Pharmaceutical (Hainan) Co., Ltd.; batch no. 6949384100644).

### 2.3. Reagents and Instruments

The following kits were used in the study: mouse 14,15-DHET ELISA kit (Shanghai Lianshuo Biological Technology Co., Ltd); mouse 20-HETE ELISA kit (Shanghai Lianshuo Biological Technology Co., Ltd); ONOO^−^ kit (Shanghai Lianshuo Biological Technology Co., Ltd); LDL-C kit (Nanjing Jiancheng Bioengineering Research Institute Co., Ltd); and TC kit (Nanjing Jiancheng Bioengineering Research Institute Co., Ltd).

The experimental modeling feed involved a dyslipidemia feed formula that consisted of 63.6% basal feed, 15% lard, 20% sucrose, 1.2% cholesterol, and 0.2% sodium cholate. The feed was provided by the Beijing Keaoxieli Feed Co.

Propylthiouracil (Ruji, 5 g/tablet) was obtained from Shanghai Ruji Biotechnology Co., Ltd. (batch no. 210720).

### 2.4. Modeling, Grouping, and Intervention Methods

Modeling involved 30 ApoE-/- mice and five C57BL/6J mice of the same strain that were adaptively fed for seven days. C57BL/6J mice were used for the normal group, and ApoE-/- mice were used for the model group. Using the random number table method, the 30 mice were divided: 25 were used to model PDR syndrome and dyslipidemia, and five were used to model SKYD syndrome and dyslipidemia. The 25 ApoE-/- mice with PDR syndrome and dyslipidemia were randomly selected and fed a high-fat diet for four weeks (weeks 1–4). The five ApoE-/- mice with SKYD syndrome and dyslipidemia were randomly selected and given 0.1% propylthiouracil by gavage at a dose of 10 mg/kg/d during weeks 1–4 and high-fat chow for two weeks during weeks 5 and 6.

Grouping, after successful modeling, involved random assignment into the normal group (Nor group,*n* = 5), model group (Mod group, *n* = 5), low-dose Erchen decoction group (L-ECD group, *n* = 5), medium-dose Erchen decoction group (M-ECD group, *n* = 5), high-dose Erchen decoction group ((H-ECD group, *n* = 5), control drug group (*n* = 5) and unmatched prescription-syndrome group (unmatched P-S group, *n* = 5)

**Figure 2:**
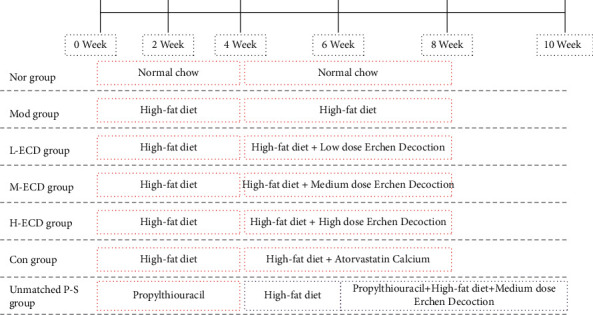
Animal modeling, grouping, and intervention flowchart.

Intervention methods involved an L-ECD group, an M-ECD group, and an H-ECD group that were gavaged with Erchen decoction dosages of 4.45, 8.90, and 17.8 g/kg/d, respectively. The control group was gavaged with an atorvastatin calcium dosage of 1.5 mg/kg/d, and the unmatched P-S group was gavaged with an Erchen decoction dosage of 8.90 g/kg/d. The 8.90 g/kg/d dosage in mice is equivalent to a 60 kg dose in adult humans, according to the human-mouse dose conversion method described in *Research Methodology of Pharmacology of Traditional Chinese Medicine*. The normal group and the model group were given the same dose of normal saline by gavage. All groups were given continuous gavage for four weeks (Figure 2).

### 2.5. Serum Sample and Aorta Sample Collection

For serum samples, after all groups were given continuous gavage for four weeks, and mice were fasted without water for 18 h before sampling and anesthesia was used for execution. Whole blood samples were taken from the animals in all groups; the samples were placed in conventional serum tubes and centrifuged at 3,000 rpm for 15 min to separate the serum. The resulting separate samples were immediately stored in a refrigerator at −80°C.

Samples of the aortic tissues from mice in the normal, model, L-ECD, M-ECD, and H-ECD groups were taken.

### 2.6. Detection of Content in Serum

For 14,15-DHET and 20-HETE determination, the 14,15-DHET and 20-HETE contents in the serum samples were measured by ELISA. The optical density was measured at a wavelength of 450 nm by an enzyme labeling instrument, and the concentration was calculated according to the standard curve.

For ONOO^−^ determination, the ONOO^−^ content in the serum samples was measured by biochemical methods, and the concentration was calculated according to the spectrophotometer findings.

For TC determination, the TC content in serum was quantified by enzyme labeling colorimetry with a kit. The optical density was measured at a wavelength of 510 nm with a microplate reader, and the concentration was calculated according to the formula.

For LDL-C determination, the LDL-C content in serum was quantified by enzyme labeling colorimetry with a kit. The optical density was measured at a wavelength of 546 nm with a microplate reader, and the concentration was calculated according to the formula.

### 2.7. Aortic Histopathology

The specimens were placed in 10% neutral formalin and fixed at room temperature for 24 h. Freshly prepared 4% formaldehyde was fixed at room temperature for 48 h and then placed into paraffin-embedded sections. The specimens were cut into 4 mm thick sections and stained with hematoxylin and eosin to observe the morphological characteristics of the vascular endothelium. Photos were taken after dyeing, and an optical microscope (AE41; Motic) equipped with a digital scanner (Panoramic MIDI; 3DHISTECH) was used to record the images of stained sections.

### 2.8. Statistical Methods

Animal-specific data were statistically processed using SPSS (version 19.0) statistical software. The measurement data were expressed as the mean ± the standard deviation. A one-way analysis of variance with randomized group design was used for comparisons between groups. Fisher's least significant difference test was used for two-way comparisons if the data variance was the same; Tamhane's test was used for two-way comparisons if the variance was not the same. Differences were considered statistically significant at *P* < 0.05. The test level was *α* = 0.05, and the confidence interval for parameter estimation was 95%.

## 3. Results

In total, 30 ApoE-/- mice and five C57BL/6J mice of the same strain without deletion were used in the experiment. Therefore, data from 35 mice contributed to the analysis of results.

### 3.1. Effects of Different Concentrations of Erchen Decoction on Serum TC and LDL-C Concentrations in Mice with PDR and Dyslipidemia (Figure 3)

After 30 days of gavage in the normal, model, L-ECD, M-ECD, H-ECD, control, and unmatched P-S groups, significant differences were noted in the serum concentration of TC (*P* < 0.05). After treatment of the P-S correspondence groups, the concentration of TC decreased to different degrees. When the serum TC concentrations were compared between groups, the L-ECD, M-ECD, H-ECD, and control group levels were all lower than those of the model group (*P* < 0.05). There was no significant difference between the concentrations in the unmatched P-S group and the model group (*P* > 0.05) (see Supplementary Material Table 1).

After 30 days of gavage in the normal, model, L-ECD, M-ECD, H-ECD, control, and unmatched P-S groups, significant differences were noted in the serum concentration of LDL-C (*P* < 0.05). After treatment of the P-S correspondence groups, the serum LDL-C concentrations were compared. When compared between groups, the concentration in the L-ECD group was lower than that of the model group (*P* < 0.05). There was no significant difference between the concentrations in the unmatched P-S group and the model group (*P* > 0.05) (see Supplementary Material Table 2).

### 3.2. Effects of Different Concentrations of Erchen Decoction on Serum ONOO^−^ Concentration in Mice with PDR and Dyslipidemia (Figure 4)

After 30 days of gavage in the normal, model, L-ECD, M-ECD, H-ECD, control, and unmatched P-S groups, significant differences were noted in the serum concentration of ONOO^−^ (*P* < 0.05). After treatment of P-S correspondence groups, the concentration of ONOO^−^ decreased to different degrees. When the serum ONOO^−^ concentrations were compared between groups, the concentrations in the L-ECD, M-ECD, and H-ECD groups were all lower than the concentrations in the model group (*P* < 0.05). There was no significant difference between the concentrations in the unmatched P-S group and the model group (*P* > 0.05) (see Supplementary Material Table 3).

### 3.3. Effects of Different Concentrations of Erchen Decoction on Pathological Staining of Aorta in Mice with PDR and Dyslipidemia (Figure 5)

Hematoxylin and eosin staining was performed on the aorta samples from mice in the normal, model, L-ECD, M-ECD, and H-ECD groups. No obvious lipid deposition or lipid stripes were found in any group; this result was in line with the characteristics of dyslipidemia models. The aortic endothelium of mice in the normal group was continuous and complete; in addition, the structure was clear, and there was no stenosis in the vascular lumen. The aortic endothelium of mice in the model group was continuous and complete, with a little irregular thickening, an unclear structure, local rupture, and inflammatory cell infiltration. The endothelium of mice in the L-ECD group showed little inflammatory cell infiltration and local fracture. The aortic endothelium of mice in the M-ECD and H-ECD groups was continuous and complete. In addition, the endothelial structure was clearer than that of the model group; the surface was smooth; and the intima, media, and adventitia were clearly displayed.

### 3.4. Effects of Different Concentrations of Erchen Decoction on Serum 14,15-DHET Concentration in Mice with PDR and Dyslipidemia (Figure 6)

After 30 days of gavage in the normal, model, L-ECD, M-ECD, H-ECD, control, and unmatched P-S groups, significant differences were noted in the serum concentration of 14,15-DHET (*P* < 0.05). After treatment in the L-ECD, M-ECD, and H-ECD groups, the concentration of 14,15-DHET increased to different degrees. When the serum 14,15-DHET concentrations were compared between groups, the levels in the normal, M-ECD, and H-ECD groups were all higher than those of the model group (*P* < 0.05); the concentration in the H-ECD group was significantly higher than that of the L-ECD group and the control group (*P* < 0.05). There was no significant difference between the concentrations in the unmatched P-S group and the model group (*P* > 0.05) (see Supplementary Material Table 4).

### 3.5. Effects of Different Concentrations of Erchen Decoction on Serum 20-HETE Concentration in Mice with PDR of Dyslipidemia (Figure 6)

After 30 days of gavage in the normal, model, L-ECD, M-ECD, H-ECD, control, and unmatched P-S groups, significant differences were noted in the serum concentration of 20-HETE (*P* < 0.05). After treatment in the L-ECD, M-ECD, and H-ECD groups, the concentration of 20-HETE decreased to different degrees. When the serum 20-HETE concentrations were compared between groups, the levels in the normal, model, L-ECD, control, and unmatched P-S groups were all higher than the level of the M-ECD group (*P* < 0.05); the concentration in the H-ECD group was significantly lower than that of the control group (*P* < 0.05). There was no significant difference between the concentrations in the unmatched P-S group and model group (*P* > 0.05) (see Supplementary Material Table 5).

## 4. Discussion

Dyslipidemia can lead to endothelial dysfunction, which is a risk for cardiovascular diseases and an early step in the clinical manifestations of atherosclerosis [14]. Severe atherosclerosis is often found in patients with coronary heart disease and dyslipidemia [15]. Gained experience in TCM treatment has proven the efficacy of TCM formulas for dyslipidemia. However, accurate syndrome diagnosis is essential to ensure the therapeutic effects of TCM in this disease.

Macrophages are a pivotal determinant of vascular endothelial injury and are indispensable for the formation of atherosclerosis [16]. We previously conducted transcriptome analysis of endothelial macrophages from mice with dyslipidemia and PDR or SKYD to identify differentially expressed genes (DEGs) in the two syndromes; we then performed Gene Ontology enrichment analysis for the identified DEGs. We found that CYP450 pathways of arachidonic acid metabolism were the primary biological process enriched in these DEGs and that dyslipidemia with PDR was often characterized by increased levels of CYP450 metabolites of arachidonic acid.

Metabolism of arachidonic acid into epoxyeicosatrienoic acid (EET) and 20-hydroxyeicosatetraenoic acid (20-HETE) by CYP450 epoxygenase [17] are associated with oxidative stress. CYP450-mediated arachidonic acid metabolism has been proven to be a feasible target in the treatment of cardiovascular diseases [18], so we explored the characteristics of dyslipidemia with PDR from this aspect. EETs are active endogenous molecules that protect against apoptosis, inflammation, and oxidation [19]; however, they are unstable in the body and are readily metabolized into dihydroxyeicosatrienoic acid by soluble epoxide hydrolase [20]. Because 14,15-EET is the most abundant EET regioisomer, we measured its concentrations to reflect EET levels [21]. Higher 20-HETE levels may aggravate oxidative stress and vascular endothelial dysfunction. Much evidence has shown that enhancing EET biosynthesis while inhibiting EET hydrolysis [22] and 20-HETE biosynthesis can ameliorate endothelial dysfunction [23].

ECD is a classic TCM formula historically recorded as a treatment for phlegm and fluid retention or as a standard treatment for phlegm. It exerts spleen-strengthening, phlegm-dispelling, and spleen–stomach qi-promoting effects that correspond to the characteristics of dyslipidemia with PDR. ECD has been applied frequently in dyslipidemia treatment [24]. The treatment has been shown to alleviate inflammation in mice with hyperlipidemia [25] and to improve lipid metabolism in rats on high-fat diets [26]. However, the effects of ECD on CYP450 pathways of arachidonic acid metabolism in dyslipidemia with PDR remain uncertain and are worth additional exploration.

Prescription-syndrome correspondence is a sophisticated approach for syndrome studies. It stresses that a matched formula can be prescribed only when the syndrome is confirmed, which offers an effective theoretical basis for the objectification of TCM syndromes. Prescription-syndrome correspondence can be evidenced by an adequate response to the representative TCM formula after treatment. Upon counterevidence for prescription-syndrome relationships, the recovery of key indicators rescued by the formula can be considered the potential material basis of the syndrome. Therefore, prescription-syndrome correspondence is a critical method for elucidating potential syndrome pathogenesis.

TC and LDL-C are the main indicators of blood lipid. The concentrations of TC and LDL-C in P-S correspondence's M-ECD group were lower than those in the control group. These results showed that ECD can improve blood lipid indicators of dyslipidemia, which was consistent with previous research conclusions [26]. Hematoxylin and eosin staining showed that no lipid deposition was present in the vascular endothelium of the animal model of the disease and syndrome, which illustrated that the model was in line with the characteristics of dyslipidemia. A comparison of the model group with the ECD groups showed that ECD can improve the structure of the vascular endothelium.

ONOO^−^ has strong oxidizing characteristics. It promotes the release of inflammatory factors through protein oxidation [27]. It causes oxidative stress and tissue injury in the vascular endothelium, which aggravates the endothelial injury. The damage is induced by promoting the aggregation of oxidized LDL, resulting in dysfunction of the cell membrane, structural damage to the cell membrane, and the formation of foam cells [28]. After treatment with ECD, the concentration of ONOO^−^ decreased to different degrees in each group. The results showed that Erchen decoction can reverse the oxidative stress index of dyslipidemia phlegm turbidity suppression syndrome. The corresponding prescriptions and syndromes showed that the enhancement of oxidative stress was a characteristic of PDR syndrome with dyslipidemia, and this determination was consistent with previous research by our group. The current study also explored the ways in which ECD reversed the effect of oxidative stress.

14,15-EET is generated from arachidonic acid metabolism and functions as a bioactive mediator [29]. 14,15-DHET, a stable metabolite of 14,15-EET, reflects EET concentrations in the circulation. The metabolite helps improve vascular endothelial function by increasing eNOS protein expression. Our results showed that 14,15-DHET concentrations significantly increased in matched P-S groups, indicating that ECD offers protection to the vascular endothelium in dyslipidemia with PDR by elevating serum 14,15-DHET. According to TCM prescription disciplines, spleen-strengthening and damp-clearing properties of *Poria cocos* perfectly correspond to endogenous phlegm-damp generation as a result of dysfunction of the spleen, which governs transportation and transformation in transporting body fluids to the entire body. Chinese plums can produce saliva and clear dryness to alleviate damage to body fluids from other drugs. Ginger can warm the spleen and stomach and dispel cold. Its warm property helps remove phlegm and strengthen spleen–stomach qi to promote transportation, also clearing phlegm. As such, TCM theory explains the potential mechanisms for the protective effect of ECD on the vascular endothelium (i.e., strengthening healthy resistance).

Our previous research on serum metabolomics of patients with PDR syndrome and dyslipidemia showed the following: the main feature of the disease syndrome is that the accumulation of harmful metabolites leads to the enhancement of oxidative stress and damage to the vascular endothelium. Increased endothelial damage caused by increased oxidative stress is a characteristic of dyslipidemia with PDR syndrome. Therefore, changes in oxidative stress-related noxious factors in mice with PDR syndrome and dyslipidemia after treatment can help explain the possible material basis of the syndrome. In our previous studies, we reported that dyslipidemia with PDR is characterized by endothelial damage induced by the accumulation of harmful metabolites, and we observed that those metabolites were altered after treatment; this finding gave insights into the possible material basis behind the syndrome pathogenesis [30].

20-HETE is a significant metabolite of arachidonic acid that is critical for the occurrence and progression of cardiovascular diseases. It is a potent vasoconstrictor that fuels vascular inflammation by increasing adhesion molecules and inflammatory cytokines induced by endothelial cell activation, and it triggers endothelial proinflammatory activation to promote atherosclerosis and vascular remodeling [31]. 20-HETE stimulates oxidative stress and a proinflammatory response by activating the nicotinamide adenine dinucleotide oxidase family in endothelial cells. Our findings showed that 20-HETE concentrations markedly decreased in the matched P-S groups versus the control group. These findings suggest that ECD inhibits 14,15-DHET expression to lower its circulating concentration, thus limiting the endothelial damage in dyslipidemia with PDR. In TCM theory, phlegm stagnation in the body may result in blood stasis in vessels, ultimately damaging arteries and veins. Among ECD components, *Pinellia ternata* takes the action of drying upon its pungent and warm properties, so it has strong phlegm-reducing power to clear phlegm stagnation and reopen vessels. The phlegm may obstruct qi flow and lead to phlegm–qi stagnation, which may cause vessel obstruction when it is prolonged. Citri reticulatae pericarpium promotes qi flow to resolve phlegm, thus smoothing qi circulation. The persistent presence of phlegm in the body also causes blood stasis. *Poria cocos* can drain and excrete water-damp to prevent phlegm retention. These are potential mechanisms to explain the effect of ECD against endothelial injury (i.e., by driving out pathogenic factors) in TCM.

Atorvastatin calcium is a classic agent against dyslipidemia; it inhibits cholesterol biosynthesis and lowers plasma LDL-C by inhibiting HMG-CoA reductase activity [32]. This study revealed that atorvastatin calcium failed to ameliorate serum 20-HETE and 14,15-DHET concentrations but did improve TC levels in mice with dyslipidemia and PDR. These findings are consistent with those of related mechanistic studies [33].

Notably, the recovery of serum TC, LDL-C, ONOO^−^, 20-HETE, and 14,15-DHET concentrations after ECD treatment in the unmatched P-S group was not as satisfactory. Treatment based on syndrome differentiation is a unique theory and clinical approach of TCM. We treat the disease, syndrome, and formula as an integral whole that has multiple intraconnections; of these, syndrome types are critical whereby a particular formula can be linked to certain diseases. For mice with dyslipidemia and SKYD in the unmatched P-S group, formulas with spleen–kidney strengthening effects are considered matched treatments; other treatments, such as ECD, cannot achieve optimal efficacy. Thus, prescription-syndrome correspondence underlies the efficacy of ECD and scientifically validates the treatment according to syndrome differentiation.

CYP450 epoxygenase may inhibit monocyte inhibition, thus shifting macrophage polarization. As part of the CYP450 epoxygenase-EET-sEH-DHET system, it exerts the biofunctions of CYP450 [34]. Physiologically, the two antagonistic molecules, EET and 20-HETE, maintain endothelial cell equilibrium. Their antagonistic relationship is supported by findings from our previous study of macrophages using RNA sequencing, which showed that DEGs in endothelial macrophages of dyslipidemia with PDR were associated with both harmful and protective biological processes. Thus, we speculate that ECD can activate macrophage polarization via CYP450 pathways of arachidonic acid metabolism, thus alleviating damage to the vascular endothelium and exerting protective effects in dyslipidemia with PDR. From the perspective of prescription-syndrome correspondence, enhanced oxidative stress via activation of endothelial macrophage polarization can be a material basis for dyslipidemia with PDR.

The main limitations of this study are as follows: the specific mechanism of action of ECD is not clear because of the small number of detection indicators. Additional studies on the specific mechanism of action and different doses or duration of Erchen decoction treatment should be carried out in the future.

## 5. Conclusion

**Figure 7:**
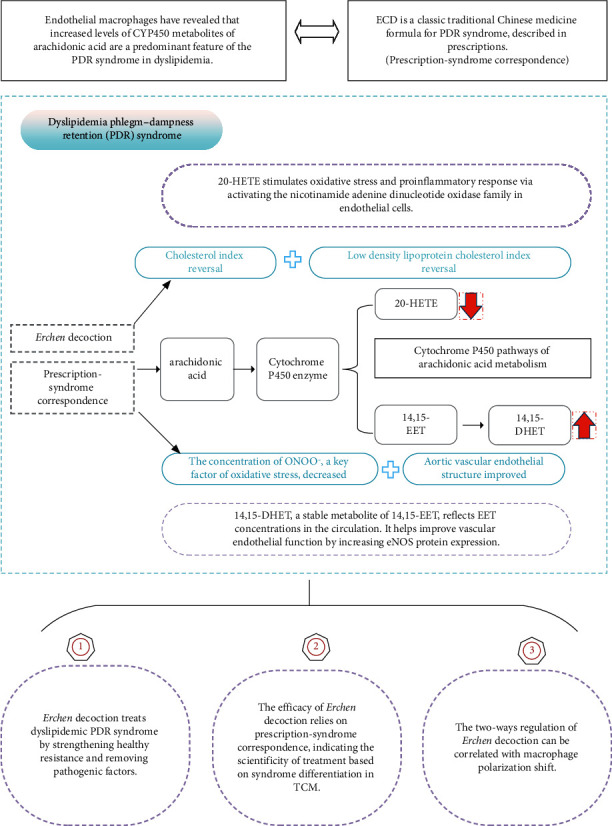
Effects of Erchen decoction on oxidative stress-related cytochrome P450 metabolites of arachidonic acid in mice with dyslipidemia and phlegm-dampness retention syndrome.

This study showed that ECD can reverse blood lipid indexes, reduce concentrations of ONOO^−^, and improve oxidative stress-related metabolite levels (elevating serum 14,15-DHET and lowering serum 20-HETE) in mice with dyslipidemia and PDR, probably via CYP450 pathways of arachidonic acid metabolism. By strengthening healthy resistance and removing pathogenic factors, ECD protects the vascular endothelium to exert therapeutic effects in dyslipidemia with PDR. Compared with unmatched P-S treatment, treatment with ECD was efficacious because of its prescription-syndrome correspondence, which thus scientifically validates the treatment according to the syndrome differentiation in TCM. When reviewed along with our previous findings, this study shows that the efficacy of ECD can be correlated with macrophage polarization. We speculate that dynamic macrophage polarization switch may be a critical process in the pathogenesis of dyslipidemia with PDR according to the prescription-syndrome correspondence theory (Figure 7).

## Figures and Tables

**Figure 3:**
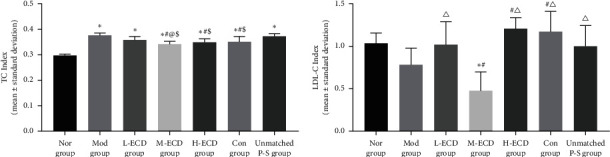
Effects of different concentrations of Erchen decoction on serum TC and LDL-C concentrations in mice with dyslipidemia and phlegm-dampness retention (PDR) syndrome. ^*∗*^*P* < 0.05 as compared to the Nor group. ^#^*P* < 0.05 as compared to the Mod group. ^@^*P* < 0.05 as compared to the L-ECD group. ^Δ^*P* < 0.05 as compared to the M-ECD group. ^$^*P* < 0.05 as compared to the unmatched P-S group.

**Figure 4:**
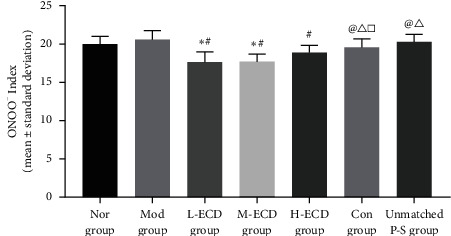
Effects of different concentrations of Erchen decoction on serum ONOO^−^ concentration in mice with dyslipidemia phlegm-dampness retention (PDR) syndrome. ^*∗*^*P* < 0.05 as compared to the Nor group. ^#^*P* < 0.05 as compared to the Mod group. ^@^*P* < 0.05 as compared to the L-ECD group. ^Δ^*P* < 0.05 as compared to the M-ECD group. ^□^*P* < 0.05 as compared to the H-ECD group.

**Figure 5:**
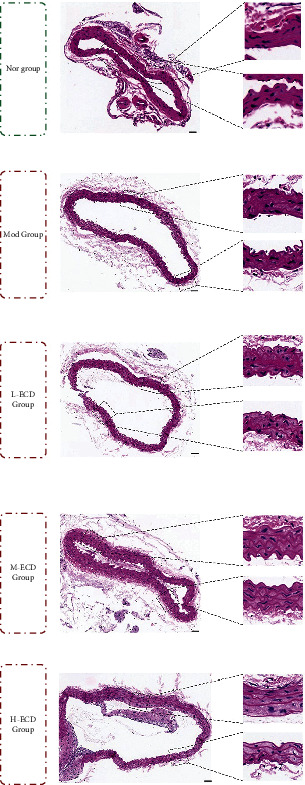
The aorta was stained with hematoxylin and eosin.

**Figure 6:**
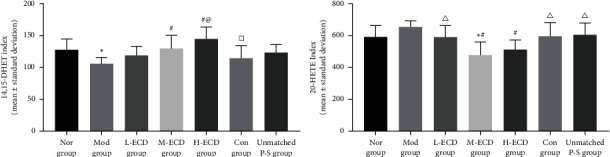
Effects of different concentrations of Erchen decoction on serum 14,15-DHET and 20-HETE concentrations in mice with dyslipidemia and phlegm-dampness retention (PDR) syndrome. ^*∗*^*P* < 0.05 as compared to the Nor group. ^#^*P* < 0.05 as compared to the Mod group. ^@^*P* < 0.05 as compared to the L-ECD group. ^Δ^*P* < 0.05 as compared to the M-ECD group. ^□^*P* < 0.05 as compared to the H-ECD group.

## Data Availability

The datasets used and/or analyzed during the current study are available from the corresponding author on reasonable request.

## Abbreviations

Nor group: Normal group
Mod group: Model group
L-ECD group: Low-dose ECD group
M-ECD group: Medium-dose ECD group
H-ECD group: High-dose ECD group
Con group: Control group
Unmatched P-S group: Unmatched prescription-syndrome
ECD: Erchen decoction.

## Supplementary Materials

Supplementary Materials The supplementary materials contain five tables. Supplementary Table 1: TC index in 7 groups of mice. Supplementary Table 2: LDL-C index in 7 groups of mice. Supplementary Table 3: ONOO^−^ index in 7 groups of mice. Supplementary Table 4: 14,15-DHET index in 7 groups of mice. Supplementary Table 5: 20-HETE index in 7 groups of mice.
