# Supplementary material for: Effects of Erchen Decoction on Oxidative Stress-Related Cytochrome P450 Metabolites of Arachidonic Acid in Dyslipidemic Mice with Phlegm-Dampness Retention Syndrome: A Randomized, Controlled Trial on the Correspondence between Prescription and Syndrome
Source: Evid Based Complement Alternat Med. 2022 Mar 29;2022:1079803. doi: 10.1155/2022/1079803 (PMC8983189; doi:10.1155/2022/1079803)
Supplement: Supplementary Materials — The supplementary materials contain five tables. Supplementary Table 1: TC index in 7 groups of mice. Supplementary Table 2: LDL-C index in 7 groups of mice. Supplementary Table 3: ONOO− index in 7 groups of mice. Supplementary Table 4: 14,15-DHET index in 7 groups of mice. Supplementary Table 5: 20-HETE index in 7 groups of mice. [file 1079803.f1.docx]

| **Table 1 TC index in 7 groups of mice (mean ± standard deviation)** | | | | |
| --- | --- | --- | --- | --- |
| **Group** | **TC index (μmol/ml)** | | | |
| Nor group | 0.297± 0.005 |  |  |  |
| Mod group | 0.377±0.009 | | | |
| L-ECD group | 0.360±0.012 | | | |
| M-ECD group | 0.344±0.010 | | | |
| H-ECD group | 0.351±0.011 | | | |
| Con group | 0.352±0.019 | | | |
| Unmatched P-S group | 0.374±0.010 | | | |
| F | 25.364 | | | |
| *P* | 0.000 | | | |

| **Table 2 LDL-C index in 7 groups of mice (mean ± standard deviation)** | | | | |
| --- | --- | --- | --- | --- |
| **Group** | **LDL-C (mmol/L)** | | | |
| Nor group | 1.035± 0.120 |  |  |  |
| Mod group | 0.787±0.193 | | | |
| L-ECD group | 1.023±0.268 | | | |
| M-ECD group | 0.481±0.220 | | | |
| H-ECD group | 1.211±0.128 | | | |
| Con group | 1.175±0.234 | | | |
| Unmatched P-S group | 1.003±0.245 | | | |
| F | 7.319 | | | |
| *P* | 0.000 | | | |

| **Table 3 ONOO^-^ index in 7 groups of mice (mean ± standard deviation)** | | | | |
| --- | --- | --- | --- | --- |
| **Group** | **ONOO^-^ (pg/ml)** | | | |
| Nor group | 20.04± 0.99 |  |  |  |
| Mod group | 20.61±1.15 | | | |
| L-ECD group | 17.69±1.30 | | | |
| M-ECD group | 16.65±0.86 | | | |
| H-ECD group | 18.97±0.88 | | | |
| Con group | 19.61±1.05 | | | |
| Unmatched P-S group | 20.33±0.94 | | | |
| F | 10.07 | | | |
| *P* | 0.000 | | | |

| **Table 4 14,15-DHET index in 7 groups of mice (mean ± standard deviation)** | | | | |
| --- | --- | --- | --- | --- |
| **Group** | **14,15-DHET index (ng/L)** | | | |
| Nor group | 128.16± 16.75 |  |  |  |
| Mod group | 106.04±9.57 | | | |
| L-ECD group | 119.60±13.63 | | | |
| M-ECD group | 130.55±20.10 | | | |
| H-ECD group | 144.87±18.96 | | | |
| Con group | 114.80±19.79 | | | |
| Unmatched P-S group | 123.77±12.67 | | | |
| F | 2.87 | | | |
| *P* | 0.026 | | | |

| **Table 5 20-HETE index in 7 groups of mice (mean ± standard deviation)** | | | | |
| --- | --- | --- | --- | --- |
| **Group** | **20-HETE index (ng/L)** | | | |
| Nor group | 593.94± 71.40 |  |  |  |
| Mod group | 655.39±38.19 | | | |
| L-ECD group | 592.69±73.07 | | | |
| M-ECD group | 478.85±81.15 | | | |
| H-ECD group | 514.40±58.14 | | | |
| Con group | 598.31±85.19 | | | |
| Unmatched P-S group | 604.86±75.84 | | | |
| F | 5.59 | | | |
| *P* | 0.009 | | | |
